# A Hybrid CPU/GPU Pattern-Matching Algorithm for Deep Packet Inspection

**DOI:** 10.1371/journal.pone.0139301

**Published:** 2015-10-05

**Authors:** Chun-Liang Lee, Yi-Shan Lin, Yaw-Chung Chen

**Affiliations:** 1 Department of Computer Science and Information Engineering, School of Electrical and Computer Engineering, College of Engineering, Chang Gung University, Taoyuan, Taiwan; 2 Department of Computer Science, National Chiao Tung University, Hsinchu, Taiwan; Universitat Rovira i Virgili, SPAIN

## Abstract

The large quantities of data now being transferred via high-speed networks have made deep packet inspection indispensable for security purposes. Scalable and low-cost signature-based network intrusion detection systems have been developed for deep packet inspection for various software platforms. Traditional approaches that only involve central processing units (CPUs) are now considered inadequate in terms of inspection speed. Graphic processing units (GPUs) have superior parallel processing power, but transmission bottlenecks can reduce optimal GPU efficiency. In this paper we describe our proposal for a hybrid CPU/GPU pattern-matching algorithm (HPMA) that divides and distributes the packet-inspecting workload between a CPU and GPU. All packets are initially inspected by the CPU and filtered using a simple pre-filtering algorithm, and packets that might contain malicious content are sent to the GPU for further inspection. Test results indicate that in terms of random payload traffic, the matching speed of our proposed algorithm was 3.4 times and 2.7 times faster than those of the AC-CPU and AC-GPU algorithms, respectively. Further, HPMA achieved higher energy efficiency than the other tested algorithms.

## Introduction

Conventional network security systems such as firewalls provide protection by inspecting packet headers for abnormal IP addresses, ports, and protocols. However, examining headers does not ensure security, especially from the perspective of application layers [1–3]; therefore, network intrusion detection systems (NIDSs) have been developed to provide greater security. A NIDS can be categorized as anomaly-based or signature-based. Anomaly-based NIDSs detect intrusions by monitoring network activity and determining if any abnormal behavior occurs [4–6]. Signature-based NIDSs determine whether incoming packet payloads contain malicious content, defined as “signatures” or “patterns.” When such patterns are found, systems generate alert messages to administrators in an effort to protect other network devices. Since signature-based NIDSs generally provide better detection against known attacks, they have been the focus of a larger number of studies.

Pattern matching is a time-consuming task for signature-based NIDSs. Previous studies [7,8] have indicated that pattern matching consumes approximately 70% of system execution time. Many software- and hardware-centered pattern-matching algorithms have been proposed for NIDSs. Hardware-based methods utilize special-purpose devices such as field programmable gate arrays (FPGAs) [9–13], content addressable memory (CAM) [14,15], and application-specific integrated circuits (ASICs) [16] to achieve high matching speeds via device parallelism. However, ASIC design and manufacturing is a time-consuming and expensive process. FPGA technology provides better flexibility than ASIC, but FPGA programming difficulties stand in the way of its widespread use [17]. In contrast, software-based methods using general-purpose processors such as the Intel x86 [18–20] provide greater flexibility and programmability. This explains in part our research focus on designing an algorithm for software-based NIDSs. The development of high-speed networks has made traditional approaches involving central processing units (CPUs) inadequate for satisfying processing speed requirements, and our goal is to take advantage of other processing units in response. Graphical processing units (GPUs), which have superior parallel processing power compared to CPUs, are likely candidates for integration with CPUs to provide high matching speeds. However, optimal efficiency cannot be achieved by sending all packets directly to a GPU for pattern-matching, due to bottlenecks associated with CPU-GPU data transmission using peripheral component interconnect express (PCIe) channels.

We propose using a hybrid CPU/GPU pattern-matching algorithm (HPMA) for deep packet inspection. HPMA uses the CPU to pre-filter incoming packets, thereby reducing the GPU workload and decreasing the potential for transmission bottlenecks. Filtered packets suspected of containing malicious content are sent to the GPU for complete matching. Data structures were designed to be sufficiently small so that most memory access requirements can be fulfilled by the CPU cache, thus enabling fast pre-filtering. Those structures also provide a high level of filtering accuracy that significantly reduces the GPU workload. We also implemented two widely used pattern-matching algorithms with both the GPU and CPU for comparison with our proposed algorithm. Test results indicate that in terms of random payload traffic, the HPMA matching speeds were 3.4 and 2.7 times faster than those of the AC-CPU and AC-GPU algorithms, respectively.

## Related Work

The literature contains numerous proposals for two kinds of pattern-matching algorithms: single-pattern and multi-pattern matching. In the first category, only one pattern can be searched at a time. Currently the Knuth-Morris-Pratt (KMP) [21] and Boyer-Moore (BM) algorithms [22] are two of the most frequently used single-pattern matching algorithms. In the second category, searches entail multiple patterns or a pattern set. Aho-Corasick (AC) [23] and Wu-Manber (WM) [24] are two well-known multi-pattern matching algorithms. Snort [25], a free and open source NIDS, used a modified version of the WM algorithm in its earlier versions. One major advantage of the WM algorithm is that it uses much less memory than the AC algorithm. However, it is sensitive to very small or large minimum pattern sizes. In contrast, the AC algorithm has deterministic worst-case performance, and is highly insensitive to pattern sets and the content being inspected. Therefore, starting with version 2.6, Snort designers adopted the AC algorithm for more comprehensive considerations.

Recent efforts to develop software-based implementations have focused on the NIDS-GPU combination. Jacob and Bordely [26] have proposed a modified KMP algorithm for integration with GPUs, and have developed a system named PixelSnort that off-loads packet processing to a GPU by encoding packets and patterns into textures. Their test results indicate that PixelSnort outperformed the well-established Snort system by approximately 40% in terms of packet processing rate, but this improvement was only evident under heavy-load conditions.

Based on the WM algorithm, Huang et al.’s [27] multi-pattern matching algorithm for NIDSs using a GPU platform contains hash functions as well as a linked list data structure. Their algorithm creates one set containing 2-byte patterns and another containing 3-byte or longer patterns. One hash function is applied to patterns in the first set, and two others are applied to the three-byte prefixes of each pattern in the second set to construct a hash space of 512x512 entries. They reported that by taking advantage of these hash functions and the high parallelism of GPUs, the processing speed of their algorithm was two times faster than that of the WM algorithm in Snort.

Vasiliadis et al.’s Gnort NIDS [28], another modification of the Snort system, uses the AC algorithm and a GPU platform to which packets are transferred for full matching, with results returned to the CPU. Gnort uses direct memory access (DMA) and the asynchronous execution of GPUs to impose concurrency between the operations handled by a CPU and GPU. Compared with PixelSnort, Gnort can achieve significant speedup for various load conditions. They reported a maximum throughput of 2.3 Gbps for synthetic traffic, and a processing speed two times faster than that of Snort for real traffic. They subsequently designed a multi-parallel intrusion detection architecture (MIDeA) that optimizes the parallel characteristics of network interface cards (NICs), CPUs, and GPUs [29]. Implemented using off-the-shelf equipment, MIDeA achieved a throughput of 5.2 Gbps under real traffic conditions.

In the above-mentioned studies, CPUs do not participate in packet processing, but only help transfer packets to GPUs. However, even though GPUs have clear advantages over CPUs in terms of parallelism, CPUs still offer great computing power. In addition, the latency of accessing packets for CPUs is shorter than that for GPUs, because GPUs need to wait for packets to be transferred from the host memory to the device memory. Therefore, cooperation between CPUs and GPUs provides a new research direction in packet matching algorithms.

According to Wu et al. [30], CPUs can cooperate with GPUs via pre-filtering. They used binary integer linear programming to generate and optimize subpattern sets from original pattern sets. Subpattern sets are loaded into the GPU device memory (also called “global memory”) where pre-filtering is performed. Results are transferred to the CPU, which proceeds with full pattern matching. However, in their paper they only describe the method of generating the subpattern set, without providing any NIDS performance results.

## The Hybrid CPU/GPU Pattern Matching Algorithm

### Notations and Problem Definition

Let *p* denote a string of characters (which can also be called a pattern) from a finite set *Λ*, and let |*p*| denote the length of pattern *p*. *P* is a set of patterns that do not contain any duplicate elements; the cardinality of any set *P* is denoted as |*P|*. Let *p*[*i*] be the *i*-th character of *p*, where 0 ≤ *i* ≤ |*p*|-1. A text *T* substring is a consecutive sequence of characters *T*[*i*]*T*[*i+1*]…*T*[*i+j*] from *T*, where 0 ≤ *i* ≤ |*T*|-1 and 0 ≤ *j* ≤ |*T*|-*i*–1. Accordingly, the pattern-matching problem can be defined as follows:

Given a text *T* and a pattern set *P*, find the largest set *S* that satisfies the conditions (a) *S* is a subset of *P* and (b) each element in *S* is a substring of *T*. For the purposes of this paper, the packet payload is treated as text *T*, as mentioned in the above definition.

### HPMA Architecture

**Fig 1:**
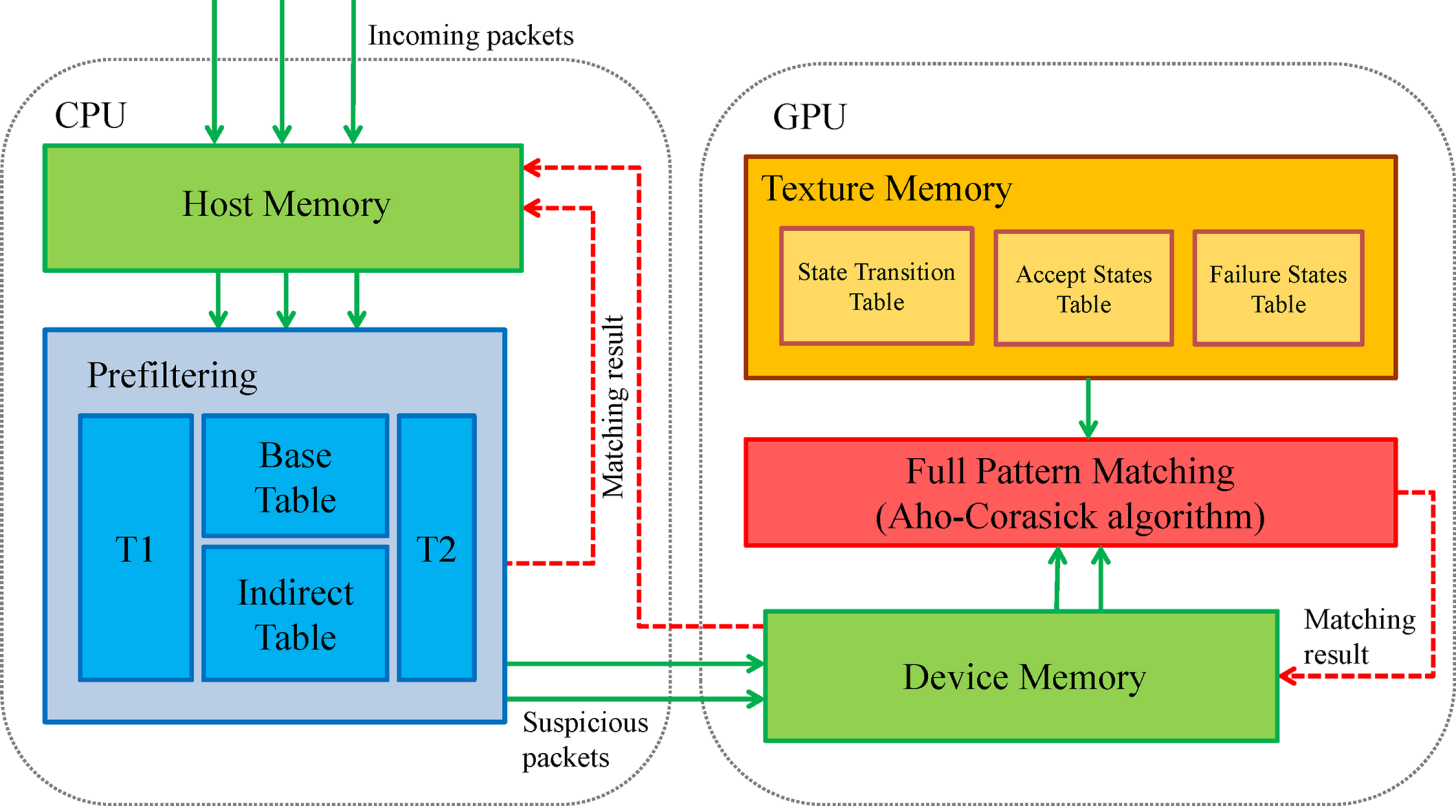
The HPMA architecture.

As shown in Fig 1, all incoming packets stored in the host memory are transmitted to the pre-filter, which operates according to four tables: *T*_1_, a *Base Table* (*BT*), an *Indirect Table* (*IT*), and *T*_2_. Table generation and pre-filtering procedures are described in detail in the following two subsections. Packets that pass through the pre-filter are guaranteed to be safe—that is, they do not contain any elements of the pattern set. Packets that are filtered out are labeled “suspicious” and transmitted to the GPU for further inspection. To reduce data transfer latency, suspicious packets are initially delivered to a buffer. All packets in a filled buffer are copied as a batch and sent to the GPU memory, where they are held until full pattern matching occurs.

The AC algorithm is implemented and ported in the GPU. All required lookup tables (i.e., state transition, accept states and failure states) are copied to the texture memory to enhance performance, since texture memory access latency is much lower than that of global memory. Full matching results are returned to the device memory and transmitted back to the CPU host memory. Note that the AC algorithm executed by the GPU can be replaced by other pattern matching algorithms. We chose the AC algorithm because it is widely used and provides the best worst-case time complexity.

### Data Structure Construction for Pre-filter

#### Frequent Two-Byte Subpattern Searches

Since most packets do not contain malicious content [31], using a single procedure to inspect every individual packet (as is the case with most pattern matching algorithms) is inefficient. We therefore tried to find a way to quickly identify non-malicious packets. Based on the simple observation that any packet containing a pattern must also contain a substring of that pattern (here we will call it a “subpattern”), a non-malicious packet must not contain any subpatterns. Let *F* denote the set of all subpatterns obtained from the pattern set. To quickly scan a packet, *F* must satisfy two conditions:

- Minimization: Since each element in *F* is a subpattern, any packet containing an element in *F* should be checked further. For this purpose, |*F*| should be as small as possible; a smaller |*F*| also reduces the required storage for pre-filtering. When the required storage size is sufficiently small, most data access requirements during pre-filtering can be fulfilled by the CPU cache, which in turn maximizes the pre-filtering processing speed.
- Regularity: All elements in *F* must be of fixed lengths so that pre-filtering can be performed efficiently.

**Fig 2:**
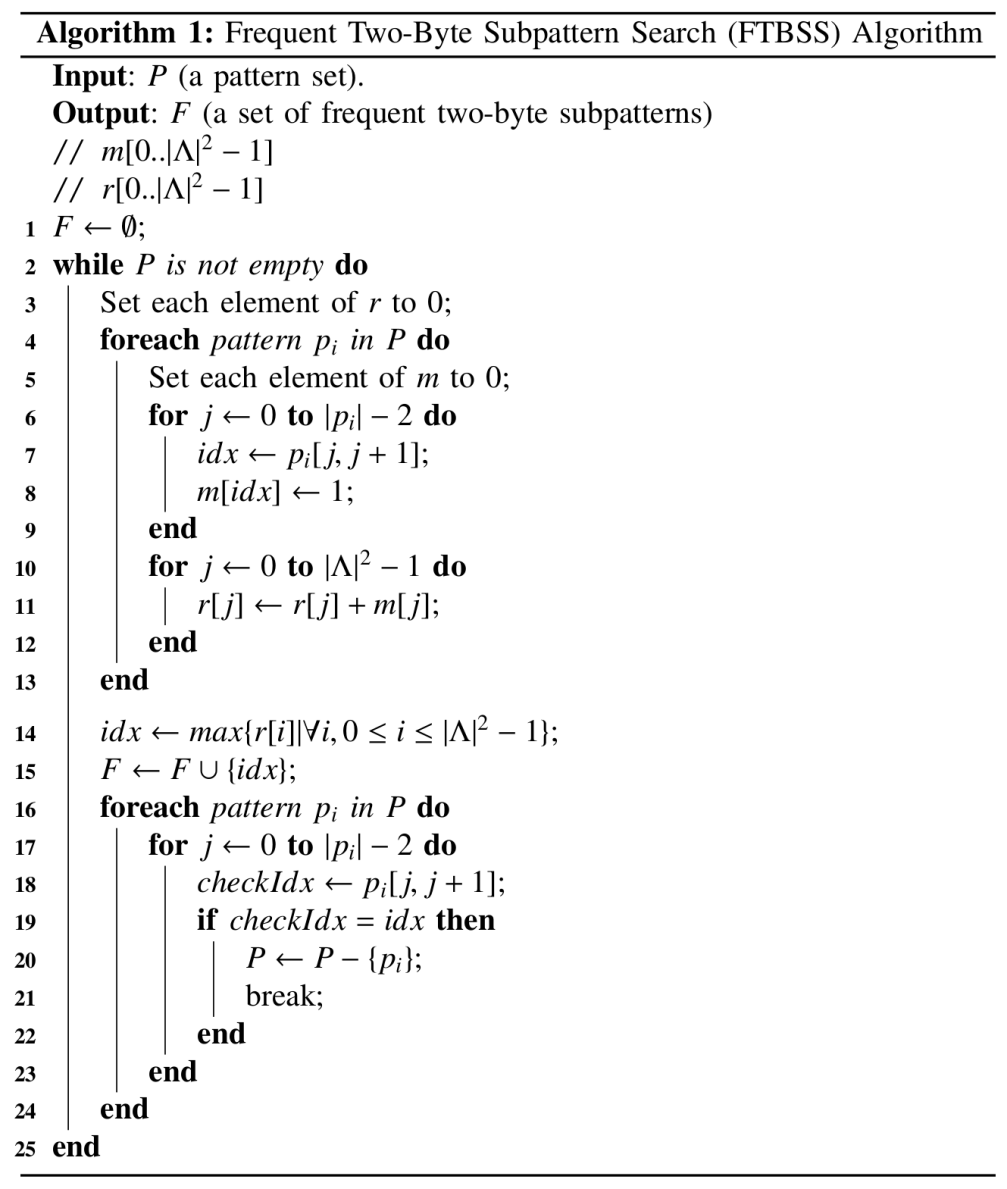
The Frequent Two-Byte Subpattern Search (FTBSS) algorithm.

To meet these two conditions, we propose using the frequent two-byte subpattern search (FTBSS) algorithm shown in Fig 2. The FTBSS algorithm takes any pattern set *P* as input and generates a subpattern set *F* in which all elements are two bytes long.

A one-dimensional array *m* is used to record all two-byte subpatterns within a pattern. Since *Λ* is the alphabet set (|*Λ*| = 2^8^), the length of *m* is |*Λ*|^2^. A one-dimensional array *r* is used to accumulate the number of times that each two-byte subpattern appears in all patterns. Initially, *F* is set as empty (line 1). The input parameter *P* initially contains all patterns. The while statement in lines 2–25 is executed when *P* is not empty—an indication that patterns require processing. The first foreach statement in lines 4–13 determines the most frequent two-byte subpattern in all patterns in *P*. For each pattern, all subpatterns serve as indexes for setting corresponding items in *m* (lines 6–9). For example, all possible two-byte subpatterns of the pattern “table” include “ta,” “ab,” “bl,” and “le”. From this point forward, digit sequences preceded by the characters 0x are considered hexadecimal integers. Since the ASCII value for “t” is 0x74 and for “a” is 0x61, 0x7461 is used as an index for setting the corresponding *m* element to 1—that is, *m*[0x7461] = 1; the respective *m* elements for the other three subpatterns are *m*[0x6162], *m*[0x626C] and *m*[0x6C65]. After processing all patterns, the *m* values are accumulated and stored in *r* (lines 10–12). Next, the most frequent two-byte subpattern is selected (line 14) and added to *F*, and all patterns containing the newly selected subpattern are removed from *P* (lines 16–24). Since the length of each element in *F* is two bytes, the FTBSS algorithm can generate sets fulfilling the above two conditions.

#### Table Construction Algorithm

**Fig 3:**
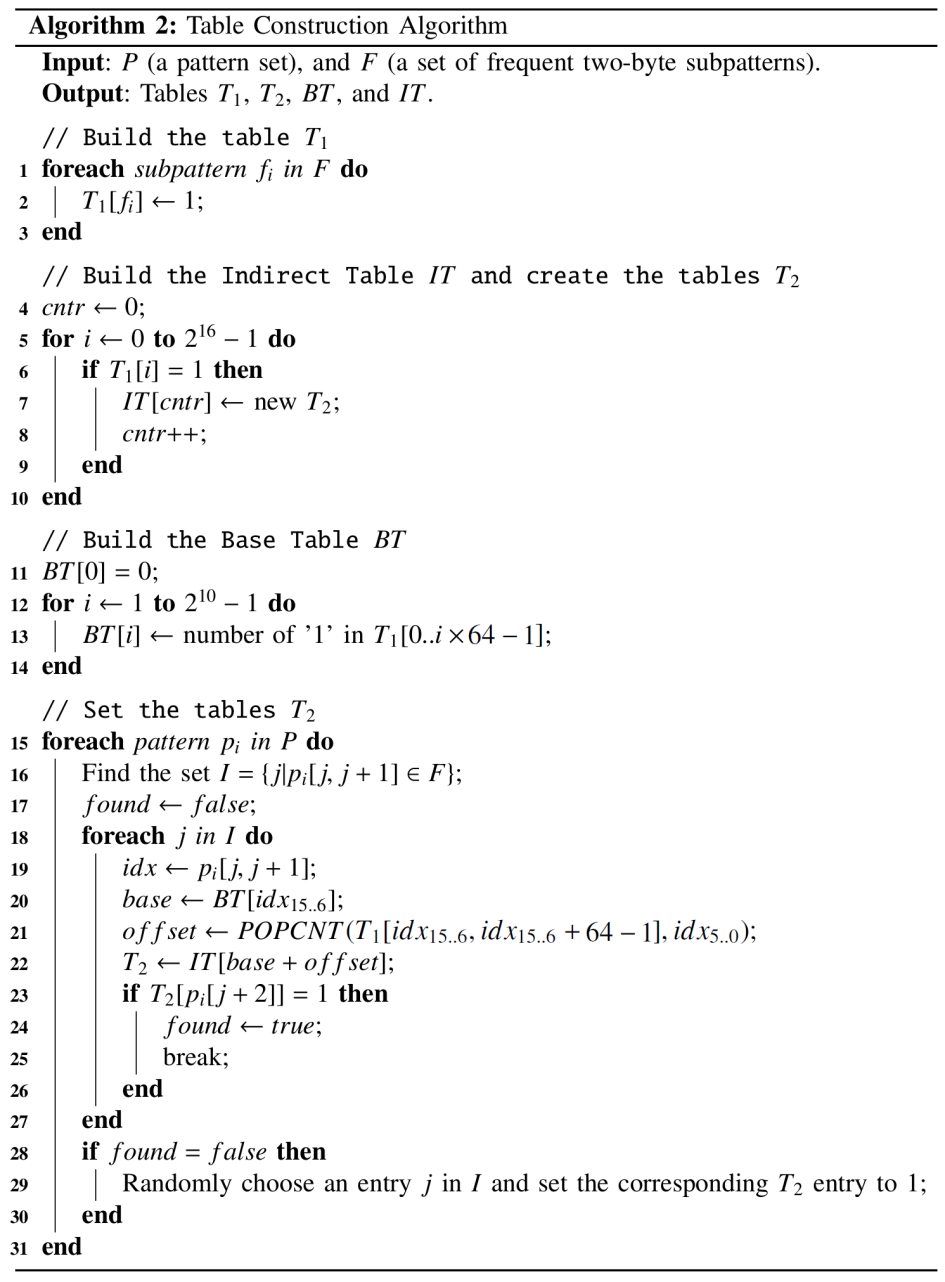
Table construction algorithm.

The second processing stage uses the pattern set and the generated two-byte subpattern set to construct the tables required by the pre-filter. As shown in Fig 3, table *T*_1_ is constructed with a 2^16^-bit array to represent *F*, with all *T*_1_ entries initially set to 0. Each two-byte subpattern can be used as a 16-bit address. For each subpattern of *F*, the corresponding *T*_1_ bit is set to 1. Accordingly, for any two successive bytes (assuming a value of *i*) in a packet payload, if *T*_1_[*i*] is 1, it indicates that the packet contains a subpattern in *F*, and therefore should be considered suspicious.

While this method has the positive characteristics of simplicity and speed, it will result in a large number of packets being delivered to the GPU for further inspection. Therefore, a method is required to reduce that number in order to achieve a higher throughput. One straightforward solution is to increase the length of subpatterns identified by the FTBSS algorithm to three bytes. Although this may be easy to do, it will increase the number of *T*_1_ entries from 2^16^ to 2^24^. Assuming that each entry requires one bit for storage, the size of *T*_1_ would become 2 MB—much larger than most L1 caches in today’s general-purpose processors, resulting in large numbers of L1 cache misses and significantly degraded throughput.

Our solution to this problem is to create three tables for reducing the memory requirements for the additional inspection byte. For each subpattern in *F*, one extra table (*T*_2_) with 2^8^ entries is used to check the next byte following the subpattern. Since the number of elements in *F* is far smaller than the number of *T*_1_ entries, it would be inefficient to store the *T*_2_ address in the corresponding *T*_1_ entry. Instead, all *T*_2_ addresses are stored in a separate indirect table (*IT*) that has a variable size depending on the number of elements in *F*. Each *T*_1_ entry only indicates whether or not the corresponding subpattern is in *F*. First, each *F* element is used to set the corresponding *T*_1_ entry (lines 1–3). A *T*_2_ table is created for each subpattern, with its starting address stored in the *IT* (lines 4–10). Suppose that a subpattern (assuming a value of *idx*) in *F* is found in a packet payload. The starting address of *T*_2_ for this subpattern can be obtained by counting the number of 1s from *T*_1_[0] to *T*_1_[*idx*], and using that number to access the *IT*.

However, the task of counting 1s is time-consuming and requires accessing half of *T*_1_ on average. We therefore propose adding a base table (*BT*) to reduce the time required to get the starting address of *T*_2_. The *i*-th entry of *BT* can be used to store the number of 1s from *T*_1_[0] to *T*_1_[i*64–1] (lines 11–14). Since *T*_1_ has 2^16^ entries, *BT* has 2^10^ entries. Assuming that the matching subpattern *p* is in the *i*-th 64-entry block of *T*_1_, the leftmost 10 bits of *p* (i.e., *i*–1) can be used to access the *BT* to retrieve the number of 1s from *T*_1_[0] to *T*_1_[(*i*–1)*64–1]. The rightmost 6 bits (assuming a value of *j*) can be used to count the number of 1s from the beginning to the *j*-th entry of the *i*-th block. Fortunately, many processors support the counting of 1s in the leading *n* bits of a 64-bit integer—for example, Intel and AMD CPUs that support Streaming SIMD Extensions (SSE) 4.2 or higher offer POPCNT instructions (which require a small number of clock cycles) for this purpose [32–34]. The combination of *BT* and POPCNT supports a solution for the counting problem that only requires one memory access and two simple calculations.

Last, each pattern in *P* is processed to create *T*_2_ tables (lines 15–31). Given that pattern *p*_*i*_ is being processed, set *I* stores the starting indexes of all elements in *F* that appear in *p*_*i*_ (line 16). Each element in *I* is then used to extract the two successive bytes from *p*_*i*_, with the obtained 16-bit value being assigned to an *idx* variable (line 19). The leftmost 10 bits of *idx* (denoted as *idx*_15..6_), which represent the number of complete 64-entry blocks of *T*_1_, can be used to access *BT* to obtain the number of 1s in the first *idx*_15..6_ 64-entry blocks (line 20). The rightmost 6 bits of *idx* (i.e., *idx*_5..0_) represent the number of bits in the last 64-entry block of *T*_1_. Function POPCNT(*x*, *y*) returns the number of 1s in the leftmost *y* bits of *x*. Thus, the number of 1s in the last 64-entry block can be obtained using POPCNT (line 21). The starting address of the corresponding *T*_2_ is consequently obtained by accessing *IT* (line 22). The byte following the two consecutive bytes being processed is used as an index to access *T*_2_. If the returned value is 1 (meaning that the corresponding *T*_2_ entry has been set to 1), then processing of the current pattern is stopped and processing of the next pattern begins (lines 23–26). If no *T*_2_ values of 1 are returned, one randomly chosen element in *I* is used to set the corresponding *T*_2_ entry to 1 (lines 28–30).

### Pre-filter Algorithm

**Fig 4:**
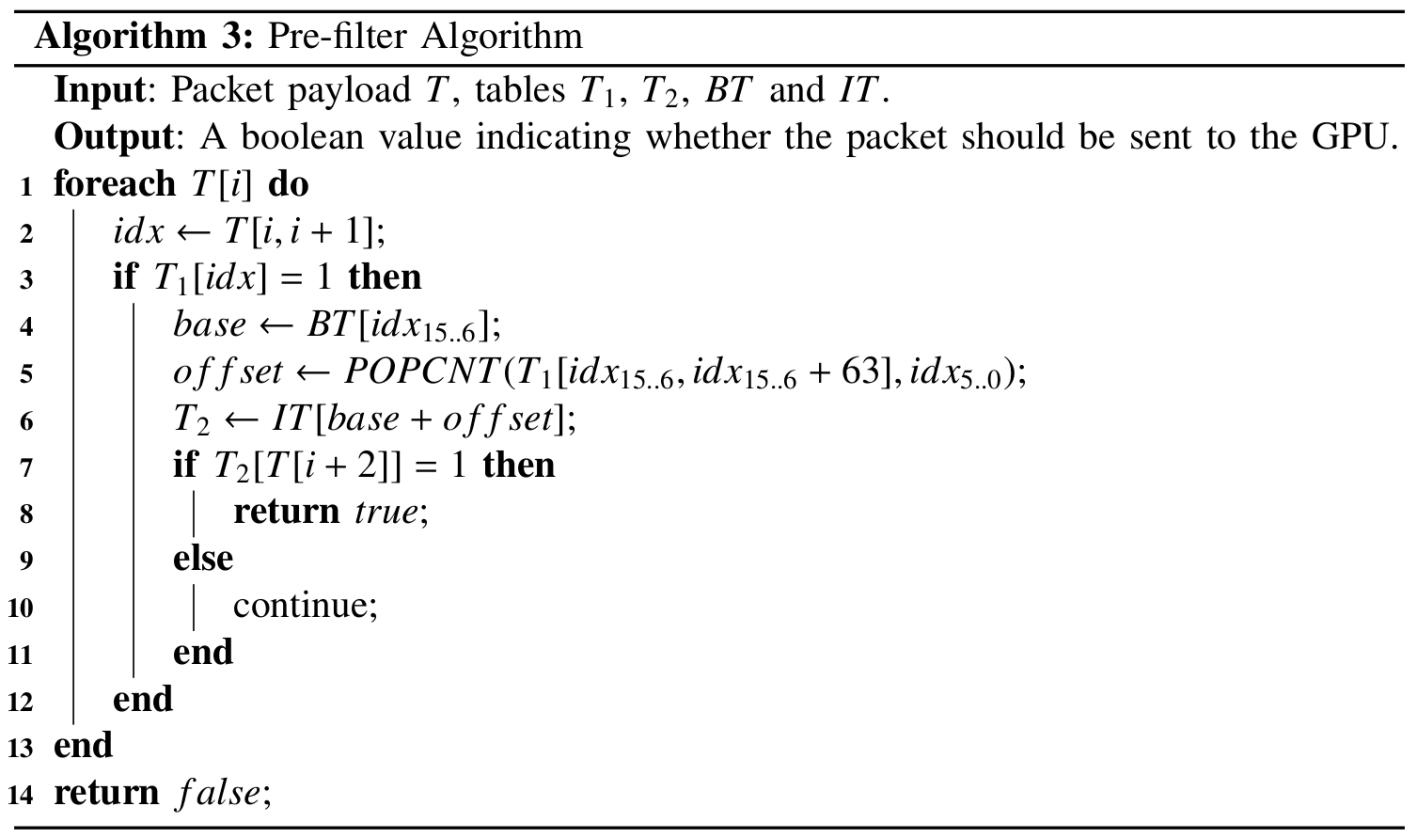
Pre-filtering algorithm.

**Fig 5:**
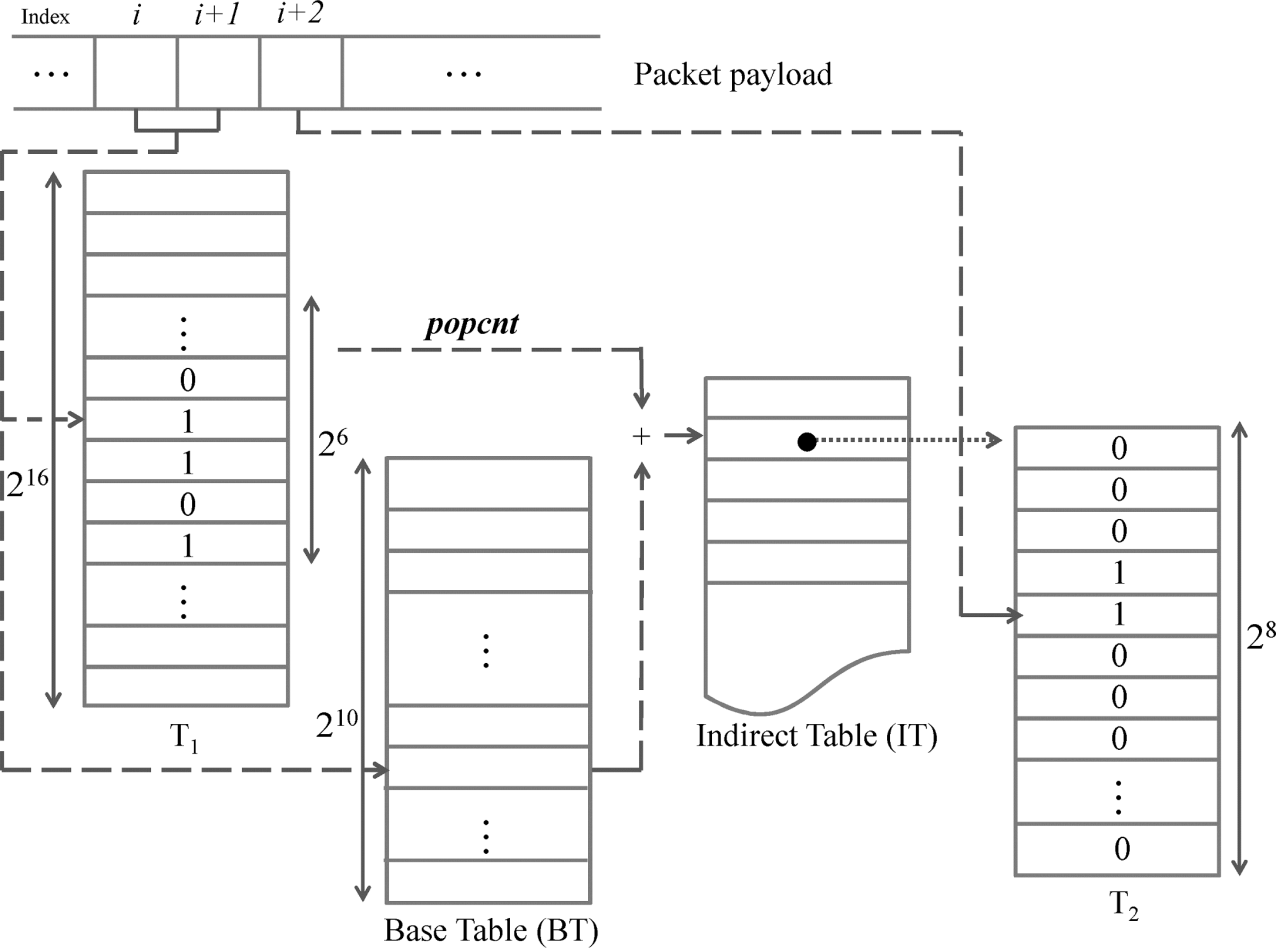
Pre-filtering procedure example.

Use of the pre-filtering algorithm shown in Fig 4 can begin once all required tables are constructed; an illustration of how the algorithm operates is shown in Fig 5. Given the current processing of the *i*-th byte of a packet payload *T*, the pre-filter obtains two successive bytes (*T*[*i*, *i*+1]; line 2) and treats them as a 16-bit index for accessing *T*_1_ (line 3). If the returned value is 0, processing of the next character begins; otherwise, the starting address of *T*_2_ is calculated using the same method shown in lines 20–22 in Fig 3. Last, the algorithm uses the byte of index *i+2* in the packet payload as the index to access *T*_2_ (line 7). If the obtained value equals 1, then the packet may contain patterns, and therefore should be identified as suspicious and sent to the GPU for full pattern matching.

## Pre-filtering Algorithm Performance Analysis

In this subsection, we analyze the probability of a randomly generated packet payload being a suspicious packet. Let *F* denote the subpattern set obtained by the FTBSS algorithm, and $p_{T_{1}}$ the probability of returning 1 when accessing *T*_1_. Since the number of entries set to 1 equals |*F*|,

$$p_{T_{1}}=\frac{\left|F\right|}{2^{16}}$$ (1)

Let |*N*| denote the average number of 1s in a single *T*_2_ table, and $p_{T_{2}}$ the probability of returning 1 when accessing *T*_2_, resulting in

$$p_{T_{2}}=\frac{\left|N\right|}{2^{8}}$$ (2)

According to the FTBSS algorithm, if accessing *T*_1_ and *T*_2_ both return 1, the packet must be sent to the GPU for further inspection. Accordingly, for the three consecutive bytes being processed, the probability of getting a match in the pre-filtering algorithm (*p*_*match*_) is

$$p_{match}=p_{T_{1}}\;*\;p_{T_{2}}$$ (3)

Since the pre-filtering algorithm simultaneously inspects three consecutive bytes, it performs a maximum of *ℓ*-2 inspections for an *ℓ*-byte packet payload. For a packet payload with a length of *ℓ* bytes, the probability of getting a match in the pre-filtering algorithm (*p*_*payload*_) can be calculated as

$$p_{payload}=p_{match}\;*\;(l-2)=(p_{T_{1}}\;*\;p_{T_{2}})\;*\;(l-2)$$ (4),

with *p*_*payload*_ dependent on both $p_{T_{1}}$ and $p_{T_{2}}$, which are affected by the pattern set. To obtain a maximum *p*_*payload*_, assume *ℓ* equals 1,460—the maximum payload size for TCP/IP packets transmitted via an Ethernet. The *best case* occurs when each pattern in *P* contains the same three-byte subpattern, meaning that both |*F*| and |*N*| = 1. Accordingly,

$$p_{payload}=(\frac{1}{2^{16}}\;*\;\frac{1}{2^{8}})\;*\;(1460-2)=0.000087$$ (5)

The *worst case* occurs in the absence of any two patterns containing the same two-byte subpattern, resulting in a maximum |*F*| value—that is, |*F*| = |*P*|. Hence, |*N*| = 1. Given 1,179 patterns in *P* (the pattern set used for performance evaluation in the next section), *p*_*payload*_ can be calculated as

$$p_{payload}=(\frac{\left|P\right|}{2^{16}}\;*\;\frac{1}{2^{8}})\;*\;(1460-2)=(\frac{1179}{2^{16}}\;*\;\frac{1}{2^{8}})\;*\;(1460-2)=0.102459$$ (6)

Best and worst cases rarely occur in practice. Using the same pattern set described in the next section, |*F*| = 301, and the 504 entries in all of the *T*_2_ tables are set to 1, resulting in

$$p_{payload}=(\frac{\left|F\right|}{2^{16}}\;*\;\frac{\left|N\right|}{2^{8}})\;*\;(1460-2)=(\frac{301}{2^{16}}\;*\;\frac{504/301}{2^{8}})\;*\;(1460-2)=0.043799$$ (7)

According to these *p*_*payload*_ calculations for different cases, the pre-filtering algorithm is capable of reducing the large number of packets that must be sent to the GPU for further inspection—only 10% of all packets in the worst-case scenario. These analytical results will be compared with experimental results in a later section.

In the previous section, we stated the inability of an initial pre-filtering algorithm to work properly with *T*_1_ only. For explanation purposes, such cases result in

$$p_{match}=p_{T_{1}}$$ (8)

$$p_{payload}=p_{match}\;*\;(l-1)=p_{T_{1}}\;*\;(l-1)$$ (9)

Given *ℓ* = 1,460, when |*F*|>45, *p*_*payload*_> 1, meaning that all packets must be sent to the GPU. In the practical case described above, |*F*| = 301 for a pattern set consisting of 1,179 patterns—evidence indicating that the pre-filtering algorithm with *T*_1_ alone cannot work in practice.

A summary of required amounts of memory for all tables used by the pre-filtering algorithm is presented as Table 1.

**Table 1:** Required amounts of memory for the pre-filter.

Table	Data size (bits)	Remarks
*T*_1_	$DS_{T_{1}}=2^{16}$	The length of each entry is 1 bit, indicating whether or not the corresponding subpattern is in *F*.
*T*_2_	$DS_{T_{2}}=\left|F\right|\;*\;2^{8}$	The number of *T*_2_ tables is |*F*|. Each *T*_2_ has 2^8^ entries.
*BT*	$DS_{BT}=\frac{2^{16}}{64}\;*\;2\;*\;8$	The length of each entry is 2 bytes.
*IT*	*DS*_*IT*_ = |*F*|*8*8	Each entry stores an 8-byte pointer to a *T*_2_
**Total**	$DS_{total}=DS_{T_{1}}+DS_{T_{2}}+DS_{BT}+DS_{IT}$	

## Experiment Evaluation

We compared HPMA performance to those of other pattern matching algorithms, especially the AC and KMP algorithms. The AC is a widely used multi-pattern matching algorithm that provides optimal worst-case time complexity. The KMP is a well-known single-pattern matching algorithm that has better worst-case time complexity than other single-pattern matching algorithms such as the BM. We tested CPU and GPU versions for each algorithm. From this point forward, AC-CPU and AC-GPU denote the CPU and GPU versions of the AC algorithm, respectively; the same notation is used for the KMP algorithm. As described above, the pattern-matching algorithm executed by the GPU is replaceable in the HPMA architecture. HPMA-AC and HPMA-KMP denote HPMA with the AC algorithm and KMP algorithm, respectively.

### Setup

Table 2 shows the hardware configuration used in our experiments, which included NVIDIA’s Compute Unified Device Architecture (CUDA) [35–38]. The pattern set was extracted from Snort rules 2008 [25]. Pattern length ranged from 1 to 208 bytes, with an average length of 13.8 bytes. Pattern set statistics are shown in Table 3. After the pattern set was obtained, random payloads with lengths of 1,460 bytes were generated. A randomly chosen pattern inserted at a random packet position served as an intrusive packet. The pattern set was inputted into the FTBSS algorithm to generate an FTBS, which was in turn inputted into the table construction algorithm to construct the *T*_1_, *T*_2_, *BT* and *IT* tables. Next, the pattern set was inputted into the AC algorithm to generate the state transition, failure state, and accept state tables.

**Table 2:** Hardware configuration for experiments.

Device	Specification
CPU	Intel Core i7 3770 @ 3.40 GHz
	Number of cores: 4
	Number of threads: 8
	Hyper threading technology
	Host momery: 8 GB DDR3
GPU	NVIDIA GeForce GTX680
	CUDA cores: 1,536
	Clock rate: 1,058 MHz
	Device memory: 2 GB GDDR5

**Table 3:** Pattern length distribution.

Pattern length	Count	Ratio
= 1	40	0.034
≦4	278	0.236
≦8	548	0.465
≦12	751	0.637
≦16	959	0.813
> 16	220	0.187
**Total count**	1179	
**Average length**	13.8	

The AC lookup tables were stored in the GPU texture memory, thus providing faster accessing speed compared to the global memory. All data discussed from this point forward are averages from 1,000 simulations. All experiments were performed on a Windows 7 Professional 64-bit OS with program priority set to “high.” To increase efficiency, the pre-filtering algorithm was implemented in a multithreaded fashion using OpenMP [39]. The Intel Core i7 CPU contains hyperthreading (HT) technology capable of using a single physical core to simulate two cores. The environment variable *KMP_AFFINITY* was set to an allocation of 8 CPU threads to enable threads 0 to 3 (T0-T3) to operate in different physical cores (Table 4). When the remaining threads were allocated, the CPU cores operated in HT mode. Processor affinity supported the use of all CPU cores. Required system processes were set to run in a single HT core, and all unnecessary background processes were closed.

**Table 4:** Thread assignments to CPU cores.

Core1	HT	Core2	HT	Core3	HT	Core4	HT
T0	T7	T1	T6	T2	T5	T3	T4

## Results and Discussion

### Impact of Number of Threads on Throughput

**Fig 6:**
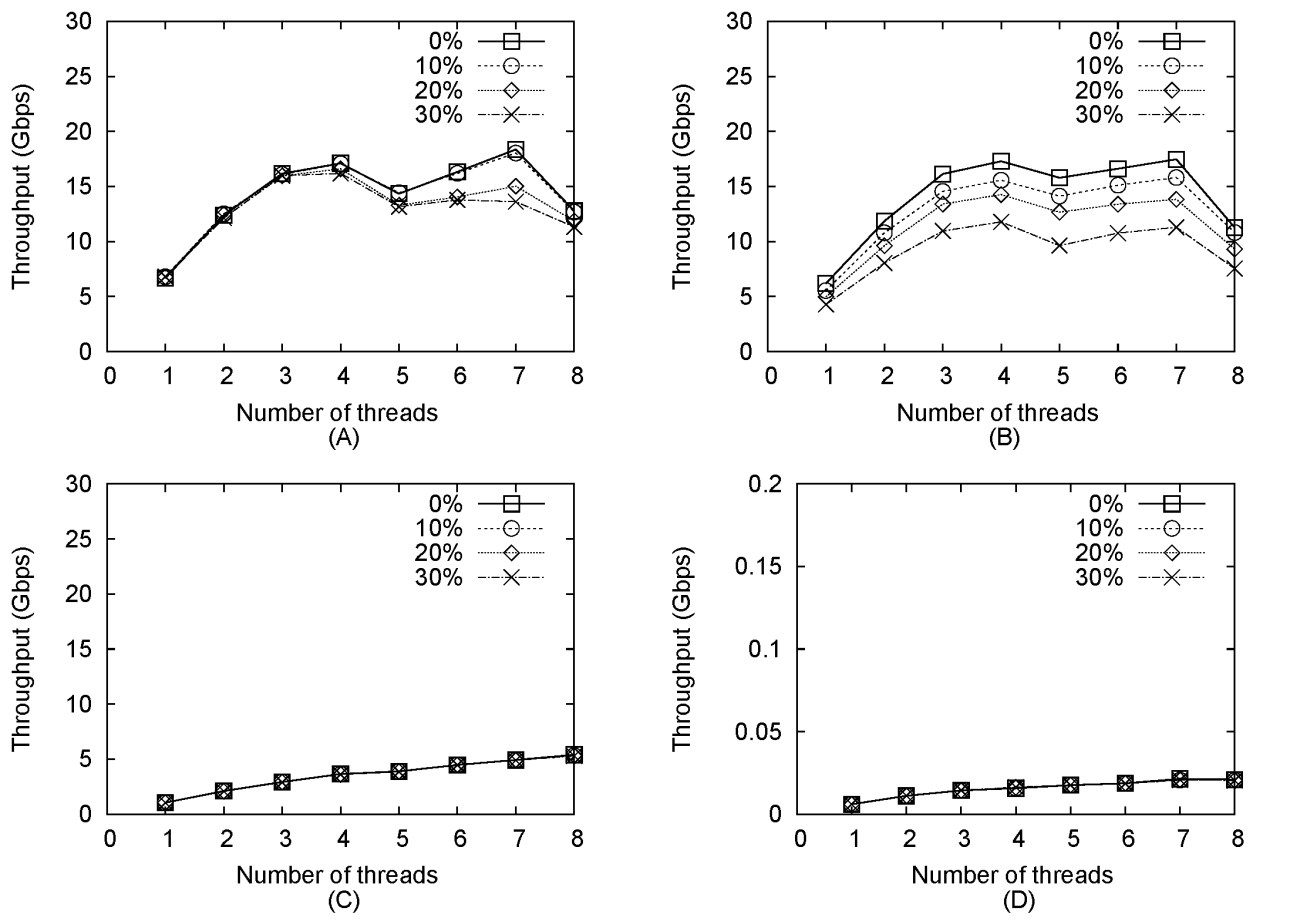
Throughput plotted against number of threads. (a) HPMA-AC (b) HPMA-KMP (c) AC-CPU (d) KMP-CPU.

To determine the relationship between throughput and intrusive packet percentage, we randomly selected a certain number of packets into which we inserted patterns randomly selected from the pattern set. As shown in Fig 6A, HPMA-AC provided a maximum throughput of 18 Gbps when seven threads were used for pre-filtering and when the percentage of intrusive packets was between 0% and 10%. When the number of CPU threads was 4 or less, HPMA-AC throughput increased as the number of CPU threads increased. However, since the CPU we used has only 4 physical cores, when the number of CPU threads exceeded 4, two HT threads executed in the same core shared the same L1 and L2 caches, increasing the potential for memory contention. Throughput continued to increase when the pre-filter operated with 6 or 7 CPU threads due to performance compensation attributed to increased parallelism, but another sharp decrease in throughput was observed when the number of CPU threads was increased to eight, likely because other system processes were being executed in one HT core, resulting in greater resource contention. We also found that the percentage of intrusive packets exerted almost no impact on HPMA-AC throughput when the number of CPU threads did not exceed 4. When 5 or more CPU threads were used, high percentages of intrusive packets (i.e., between 20% and 30%) cause the HPMA-AC throughput to decrease. This can be explained by the use of the CPU HT mode and transmission bottlenecks between the CPU and GPU.

As shown in Fig 6B, HPMA-KMP provided a maximum throughput of 17 Gbps, which was slightly lower than that of the HPMA-AC. HPMA-KMP exhibited a similar relationship between throughput and intrusive packet percentage. The main difference between HPMA-AC and HPMA-KMP was the higher HPMA-AC throughput for the same experimental parameters, indicating lower KMP efficiency for GPU execution. This also explains why the difference in throughput increased as intrusive packet percentage increased.

Throughput values for AC-CPU with different numbers of threads are shown in Fig 6C. Maximum throughput was only 5 Gbps when 8 CPU threads were used—in other words, unlike the scenario described above, performance gain was proportional to the number of CPU threads, since the tables used by the AC algorithm were much larger in size compared to the CPU cache. This means that most of the data had to be accessed from the main memory. Since main memory latency was much larger than cache latency, memory contention between HT threads exerted a minor effect.

KMP-CPU throughput values for different numbers of threads are shown in Fig 6D. Since KMP works with single patterns, all patterns must be matched one-by-one. Maximum throughput was only 21 Mbps for a total of 1,179 patterns—much lower than those of other algorithms. Similar to AC-CPU, performance gain was proportional to the number of CPU threads.

### Impact of Pattern Set Size on Throughput

**Fig 7:**
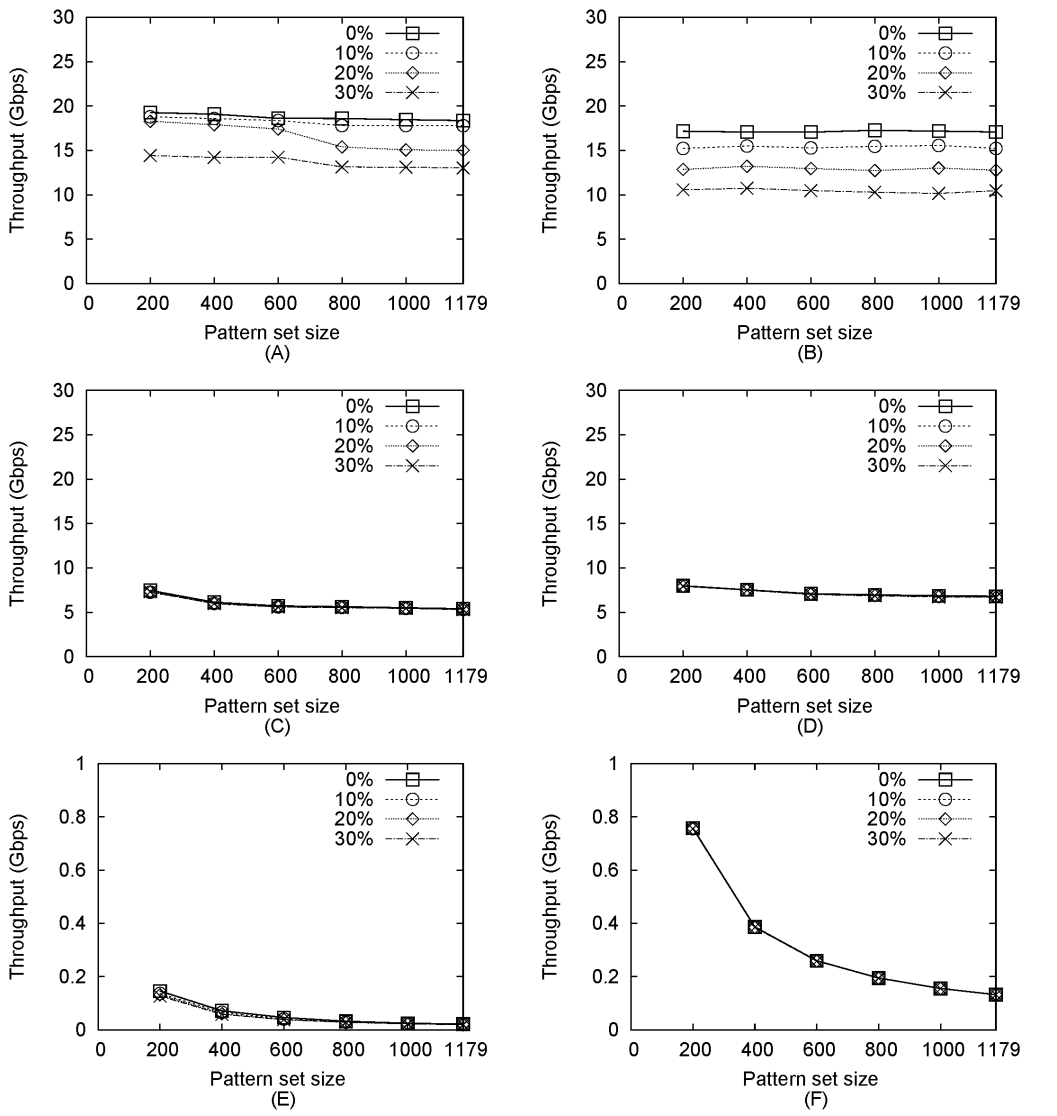
Throughput plotted against pattern set size. (a) HPMA-AC (b) HPMA-KMP (c) AC-CPU (d) AC-GPU (e) KMP-CPU (f) KMP-GPU.

Data for the effects of pattern set size on HPMA-AC, HPMA-KMP, AC-CPU, AC-GPU, KMP-CPU, and KMP-GPU algorithm throughputs are shown in Fig 7. HPMA-AC and HPMA-KMP used 7 CPU threads, while AC-CPU and KMP-CPU used 8.

As shown in Fig 7A, pattern set size exerted little impact on HPMA-AC throughput when intrusive packet percentages were 0% or 10%, but throughput dropped for sets containing more than 600 patterns when the percentage was 20%. Recall that memory size required by the pre-filter is dependent on the number of patterns; therefore, when the number exceeded 600, increased cache contention resulted in degraded throughput. At an intrusive packet percentage of 30%, transmission between the CPU and GPU was affected by a throughput bottleneck due to the large number of packets that had to be sent to the GPU for full matching. A slight difference was noted between the effects of pattern set size on HPMA-AC and HPMA-KMP. As shown in Fig 7B, it exerted no impact on HPMA-KMP throughput for various intrusive packet percentages. Since the throughput of the KMP algorithm executed by the GPU was much lower than that of the pre-filter, it was significantly affected by the number of packets that needed to be sent to the GPU—that is, throughput decreased as intrusive packet percentage increased.

As shown in Fig 7C, intrusive packet percentage exerted no impact on AC-CPU throughput, since the AC algorithm had to inspect every byte of each packet whether it was intrusive or not. The number of patterns exerted little impact on throughput—for example, 7 Gbps at 200 patterns, higher than in other cases due to the size of required storage, which increased as the number of patterns increased. For small pattern sets, the AC algorithm may achieve higher throughput levels due to the increase in memory access associated with CPU caches, which provide much shorter access latency than main memory sources. However, the AC algorithm requires a large amount of memory to store lookup tables, which explains why throughput dropped and essentially remained unchanged at 5 Gbps when the number of patterns exceeded 400. As shown in Fig 7D, only two subtle differences were observed between AC-GPU and AC-CPU throughputs. First, due to the GPU’s superior parallel computing power, AC-GPU performed better: approximately 8 Gbps throughput when the number of patterns was 200, and fixed at 7 Gbps when the number exceeded 400. Second, the impact of the number of patterns on GPU throughput was less than that for AC-CPU. Since the CPU-GPU transmission bottleneck dominated inspection overhead, the memory access latency of the GPU was less significant compared to that of the CPU cache.

As shown in Fig 7E, the effects of pattern set size on KMP-CPU throughput were similar to those for AC-CPU. However, KMP-CPU throughput was much lower than AC-CPU throughput because the KMP algorithm matched only one pattern at a time. Even for the smallest pattern set with only 200 patterns, throughput was not comparable to that for the AC-CPU algorithm. In contrast, the KMP-GPU throughput was around four times that of the KMP-CPU throughput for 200 patterns (Fig 7F). As the number of patterns increased, KMP-GPU throughput decreased proportionally.

### Overall Comparison

**Fig 8:**
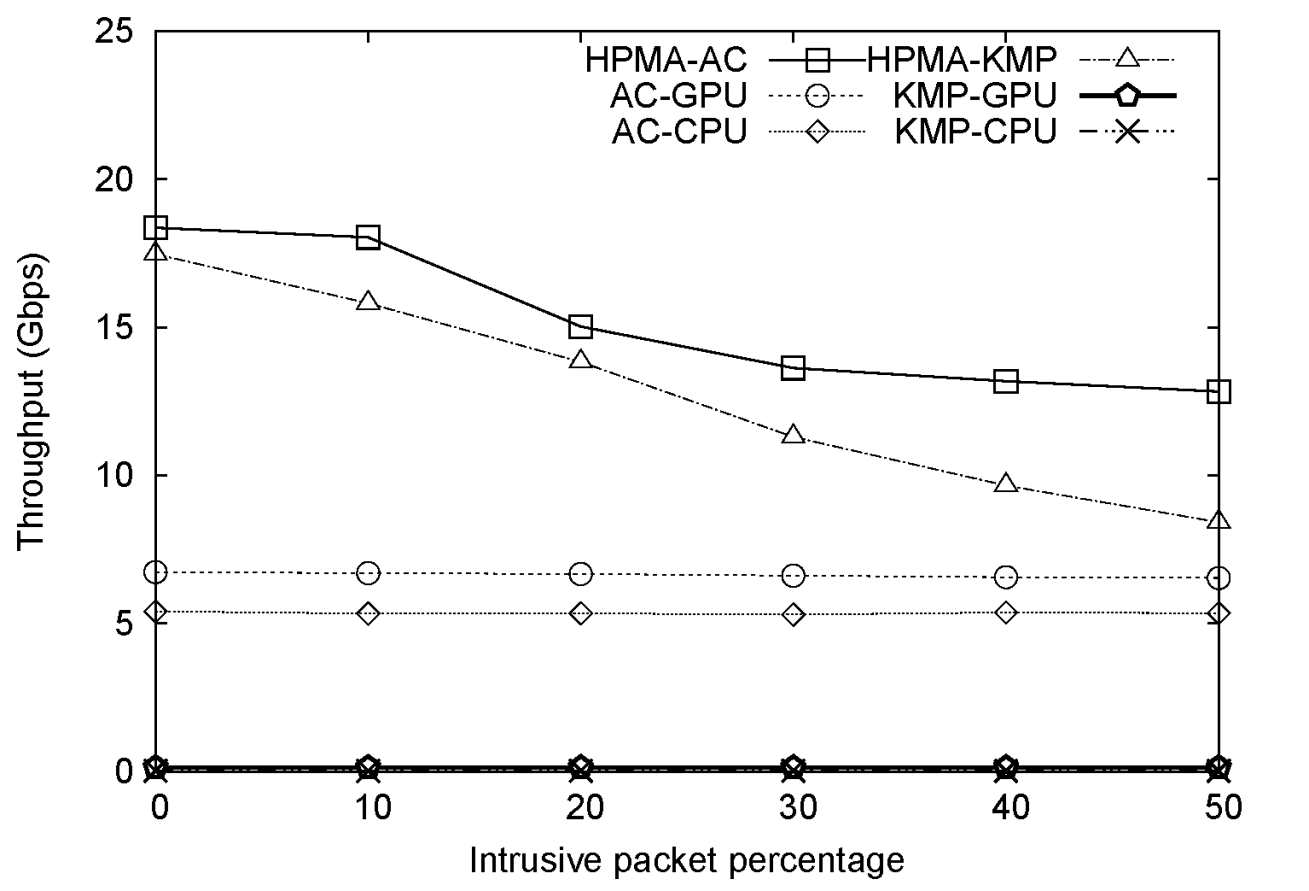
All algorithm throughput values plotted against intrusive packet percentages.

**Fig 9:**
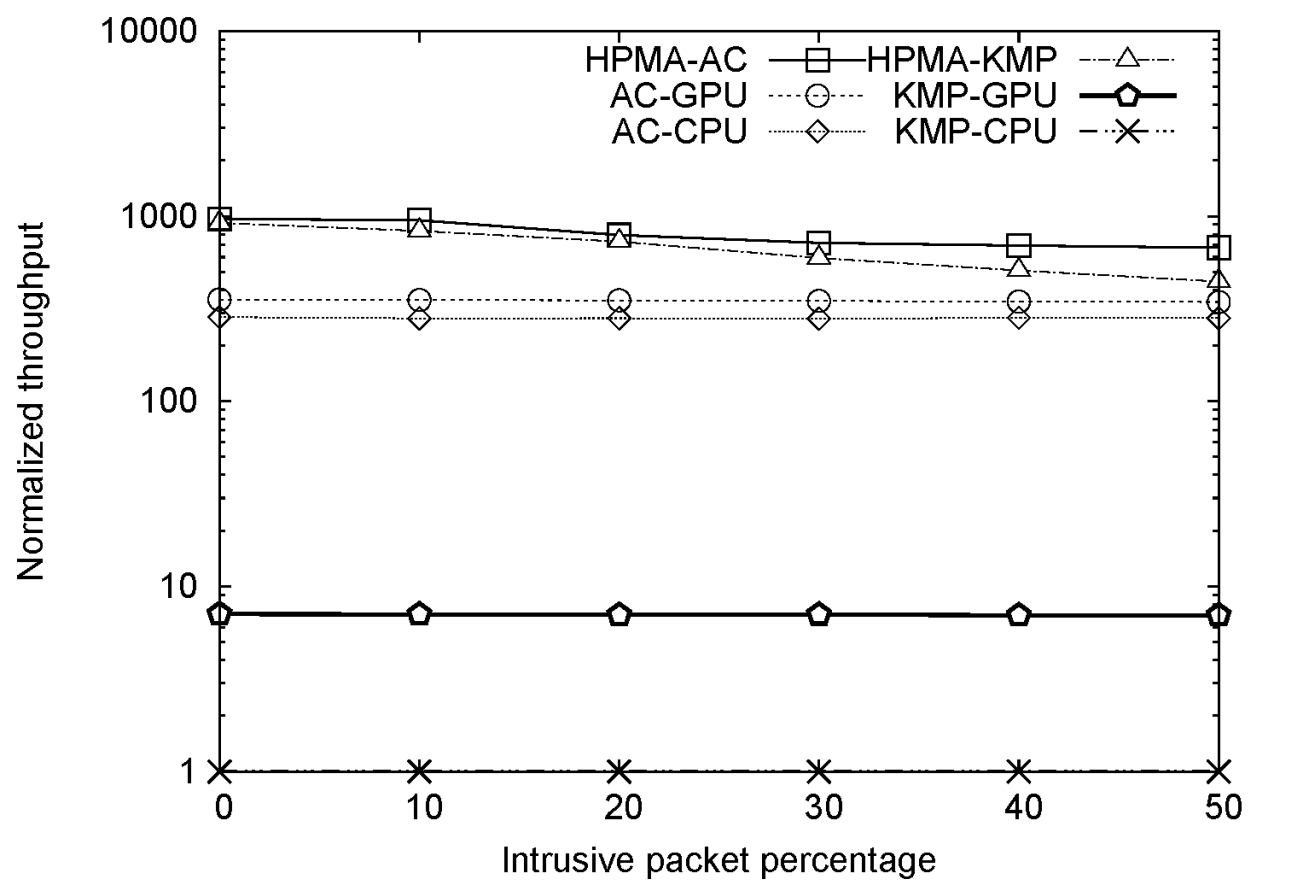
Normalized throughput values plotted against intrusive packet percentages.

A comparison of AC-CPU, AC-GPU, HPMA-AC, KMP-CPU, KMP-GPU and HPMA-KMP algorithm throughputs for different percentages of intrusive packets is presented in Fig 8 (number of patterns = 1,179). To more clearly illustrate performance improvement, normalized throughputs for all six methods with KMP-CPU used as a baseline are depicted in Fig 9. As shown in Fig 8, HPMA-AC significantly outperformed the other five algorithms, achieving a throughput of more than 18 Gbps when the percentage of intrusive packets was 10% or less. In comparison, AC-CPU and AC-GPU throughputs were 5 and 7 Gbps, respectively. As discussed above, KMP algorithm throughput is sensitive to pattern set size. Since the total number of patterns was large, KMP-CPU and KMP-GPU throughputs were only 21 Mbps and 130 Mbps, respectively. HPMA-AC throughput started to decline when 20% or more of the packets were intrusive, since the bottleneck associated with CPU-GPU transmission via the PCIe became more evident. Note that AC-CPU, AC-GPU, KMP-CPU, and KMP-GPU all achieved identical throughputs at various intrusive packet percentages. The results presented in Fig 9 indicate that HPMA-AC outperformed AC-CPU by 3.4 times and AC-GPU by 2.7 times in terms of throughput when the intrusive packet percentage was 0%; at 50%, HPMA-AC outperformed AC-CPU and AC-GPU by 2.4 times and 1.9 times, respectively.

### Pre-filter Percentages

As stated, the purpose of the HPMA pre-filter is to identify packets that might contain malicious patterns. The potential for misidentification makes it important to measure pre-filtering accuracy. Eq (4) can be used to calculate the probability of a randomly generated packet payload being a suspicious packet; we call this probability the theoretical *p*_*payload*_. Table 5 presents statistics for tables *T*_1_ and *T*_2_ and theoretical *p*_*payload*_ values for various pattern set sizes. A comparison of practical *p*_*payload*_ and theoretical *p*_*payload*_ values at various intrusive packet percentages is shown in Table 6. The data indicate a strong correspondence between the two—that is, a difference ranging from 2.2% to 4.4%. According to these results, the proposed HPMA is capable of accurately identifying and filtering out intrusive packets, thereby achieving higher throughput levels compared to other algorithms.

**Table 5:** Theoretical *p*_*payload*_ values for various pattern set sizes.

Pattern set size	Number of 1s in *T*_1_	Number of 1s in all *T*_2_s	Theoretical *p*_*payload*_
200	82	123	0.010688
400	145	212	0.018423
600	192	308	0.026766
800	239	387	0.033631
1000	277	454	0.039454
1179	301	504	0.043799

**Table 6:** Comparison of practical *p*_*payload*_ and theoretical *p*_*payload*_ values.

Intrusive packet percentage	Practical *p*_*payload*_	Theoretical *p*_*payload*_	Difference
0%	4.4108%	4.3799%	4.4%
10%	14.0439%	13.9419%	4.0%
20%	23.5171%	23.5039%	3.5%
30%	33.2161%	33.0659%	3.2%
40%	42.6680%	42.6267%	2.7%
50%	52.2408%	52.1890%	2.2%

### Required Pre-filter Memory Amount

Table 7 shows the required pre-filtering memory sizes (*DS*_*total*_) for various pattern set sizes; it is based on the results shown in Table 1. |*F*| values in each case are also presented. Note that the HPMA pre-filter used a small amount of memory—somewhere between 13.2 and 21.8 KB, making it possible for most memory access tasks to be performed using CPU caches, thereby enhancing pre-filter speed.

**Table 7:** Required pre-filtering memory sizes for various pattern set sizes.

**Pattern set size**	200	400	600	800	1000	1179
**|*F*|**	82	145	192	239	277	301
**Memory size (KB)**	13.2	15.7	17.5	19.3	20.8	21.8

### Results for Real Packet Traces

Note that the results presented above are based on randomly generated and distributed intrusive packets. Two real packet traces were used to study the impacts of realistic and intense traffic on throughput:

1. Defcon: This trace file was obtained from the Capture-the-Flag contest held at Defcon 17 [40], the world’s largest security hacking game, with competitors breaking into servers while defending their own from attacks. Accordingly, this trace file contains a large number of suspicious packets.
2. Web traffic: This trace file was obtained by generating a number of web requests to popular portal sites, including Google, Yahoo, and Amazon.

Table 8 presents statistics for the two packet traces. Experimental results for six algorithms using the Defcon and web traffic traces are shown in Table 9. For the Defcon trace, AC-CPU had the highest throughput, followed by HPMA-AC and HPMA-KMP. HPMA failed to achieve the highest throughput because the Defcon trace contained too many suspicious packets. As shown in Table 8, 76.3% of the packets (or 88.8% of all traffic) had to be sent to the GPU for full pattern matching, resulting in degraded throughput. In contrast, the web traffic trace contained a much smaller number of suspicious packets, which explains the top two rankings of the HPMA-AC and HPMA-KMP throughputs. As stated, HPMA throughput improvement was due to the pre-filter; conversely, when the pre-filter failed to make a significant reduction in the number of packets that were needed to be sent to the GPU for full pattern matching, HPMA was incapable of achieving good throughput. However, even for very high intrusive packet percentages such as those found in the Defcon trace, HPMA still achieved comparable throughputs compared to other algorithms, regardless of whether the AC or KMP algorithm was executed by the GPU.

**Table 8:** Statistics of the Defcon and web traffic traces.

	Defcon	Web traffic
Number of packets	2,048,404	390,920
Average packet length (bytes)	267.29	1,282.36
Standard deviation of packet length (bytes)	372.81	380.35
Intrusive packet percentage	23.2%	34.7%
Percentage of packets sent to the GPU	76.3%	40.7%
Percentage of traffic sent to the GPU	88.8%	34.5%

**Table 9:** All algorithm throughput values (in Gbps) using Defcon and web traffic traces.

Algorithm	Defcon17	Web traffic
AC-CPU	3.051	4.585
AC-GPU	1.125	5.919
HPMA-AC	2.258	12.153
KMP-CPU	0.019	0.021
KMP-GPU	0.031	0.104
HPMA-KMP	1.318	10.078

Note that AC-CPU throughput was higher than AC-GPU throughput for the Defcon trace, while the relationship was reversed for the web traffic trace. GPU parallelism is generally much better than CPU parallelism; therefore the GPU version throughput for a pattern matching algorithm should be higher than that of the CPU version. However, most Defcon trace packets were small–-an average packet size of only 267 bytes, with 64% less than 200 and 86% less than 400 bytes. The small packets reduced transmission efficiency between the CPU and GPU, and degraded GPU processing efficiency. In contrast, the average web traffic trace packet size was 1,282 bytes, with 82% larger than 1,200 bytes. This explains why the AC-GPU throughput was higher than the AC-CPU throughput for the web traffic trace.

### Energy Consumption Comparisons for Various Algorithms

**Fig 10:**
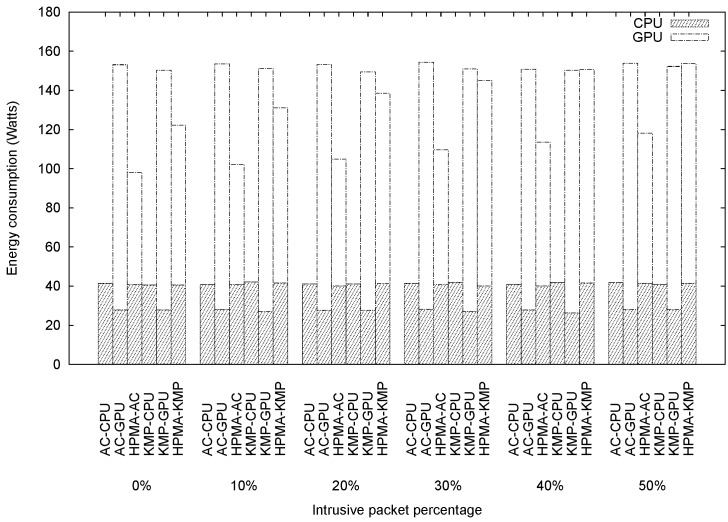
Energy consumption values plotted against intrusive packet percentages.

Since HPMA uses the CPU and GPU at the same time, it is reasonable to assume that it consumes more energy than other algorithms, raising the question of whether the throughout improvement is worth the extra energy consumption. We adopted Intel Power Gadget [41] and GPU-Z [42] to measure CPU and GPU energy consumption, respectively. Intel Power Gadget is an energy-monitoring tool for second-generation (or later) Intel Core processors. In addition to a graphical user interface (GUI), it also contains a C/C++ application programming interface (API) for developers to use in their work. The GPU-Z utility displays information about video cards and GPUs. As shown in Fig 10, the AC-CPU and KMP-CPU algorithms consumed the least energy among the six algorithms tested, which is unsurprising because they use only the CPU for pattern matching. For AC-GPU and KMP-GPU, the CPU only performed the task of transferring packets to the GPU, and therefore consumed much less energy than the other algorithms. However, since all packets were processed by the GPU, GPU energy consumption was significant (approximately 120 watts). As a result, overall energy consumption values for these two algorithms were ranked first and second for intrusive packet percentages between 0% and 30%. Energy consumption levels for HPMA-AC and HPMA-KMP were less than those for AC-GPU and KMP-GPU. Although HPMA usage did not result in reduced energy consumption by the CPU, the pre-filter successfully reduced the number of packets requiring GPU processing, thereby reducing GPU energy consumption; consumption increased as the number of packets sent to the GPU for full matching increased. Compared with HPMA-AC, HPMA-KMP consumed more energy because the KMP algorithm executed by the GPU was less efficient than the AC algorithm.

**Fig 11:**
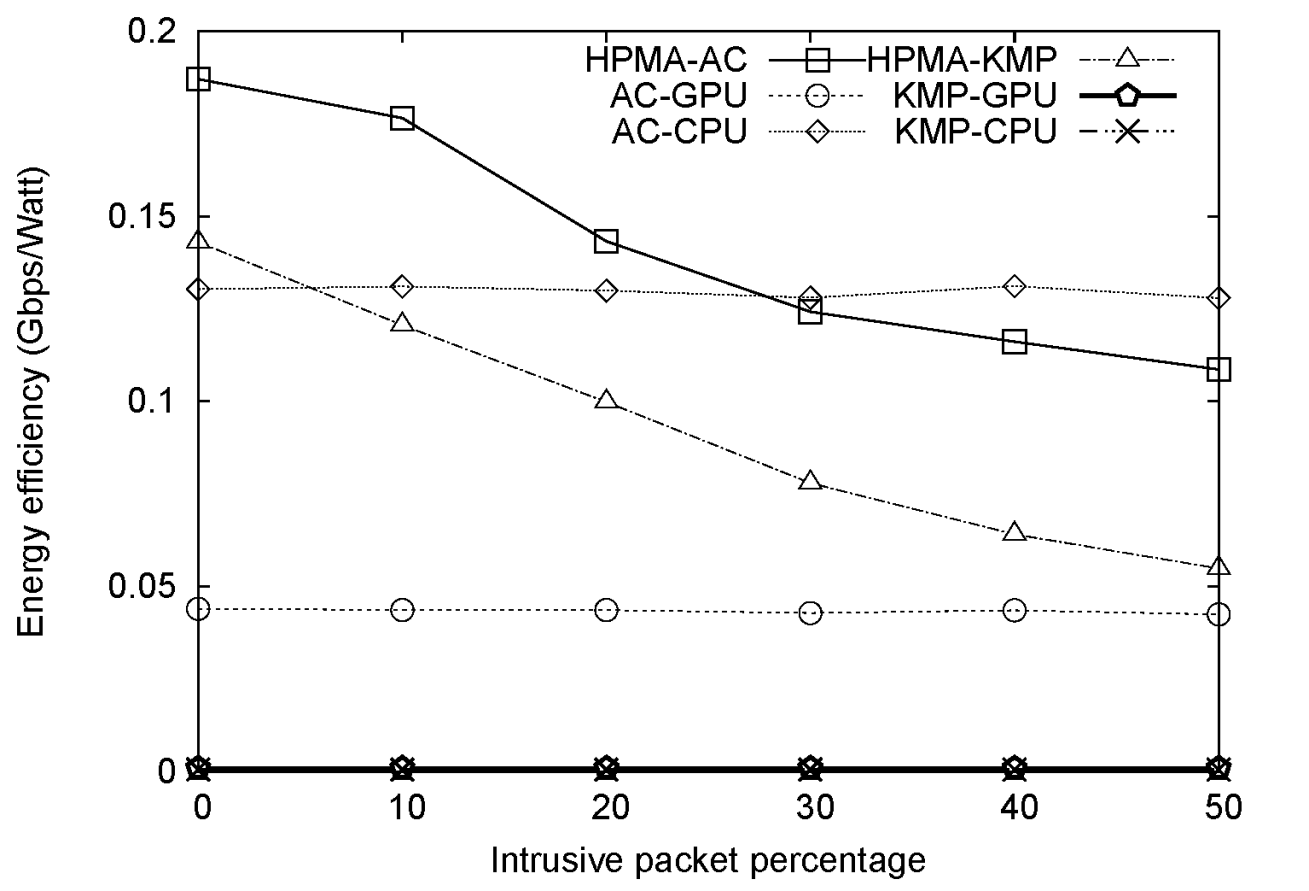
Energy efficiency values plotted against intrusive packet percentages.

Although HPMA did not consume the smallest amount of energy among the six algorithms, HPMA-AC did achieve the highest throughput. Further study is required to determine whether the tradeoff is worthwhile. The energy efficiency of a pattern matching algorithm is measured in terms of throughput per watt. As shown in Fig 11, the HPMA-AC had the best energy efficiency when intrusive packet percentages were 20% or less. Both HPMA-AC and HPMA-KMP energy efficiency decreased when intrusive packet percentages increased, while the energy efficiencies of other algorithms remained approximately the same for various intrusive packet percentages. The reason is that as the intrusive packet percentage increased, more packets had to be sent to the GPU for full matching, not only increasing GPU energy consumption, but also decreasing throughput.

## Conclusion

In this paper we described our proposal for a Hybrid CPU/GPU Pattern-Matching Algorithm (HPMA) to improve the performance of signature-based NIDSs implemented using various software platforms. A pre-filtering mechanism and data structure were designed to support appropriate workload allocation between a CPU and GPU in order to minimize GPU data delivery bottlenecks. The sufficiently small data structure generated by HPMA uses CPU caches efficiently so as to achieve faster pre-filtering speeds. In addition to describing the HPMA architecture and data structure generation, we offered theoretical data in support of results from experiments involving the effects of CPU thread allocation, the effects of input pattern set size, and performance comparisons with other inspection algorithms. According to our results, the HPMA-AC outperformed AC-CPU by 3.4 times and AC-GPU by 2.7 times in terms of inspection speed with random payload traffic, indicating enhanced efficiency due to CPU-GPU cooperation. In addition, HPMA-AC achieved higher energy efficiency than the other tested algorithms.

## References

[1] Handley M, Paxson V, Kreibich C (2001) Network intrusion detection: evasion, traffic normalization, and end-to-end protocol semantics. Proceedings of USENIX Secur Symp, 115–131.

[2] Kruegel C, Valeur F, Vigna G, Kemmerer R (2002) Stateful intrusion detection for high-speed networks. Proceedings of IEEE Symp Secur Priv, 285–293.

[3] Paxson V (1999) Bro: a system for detecting network intruders in real-time. Computer Networks 31(23): 2435–2463.

[4] Tian D, Liu YH, Xiang Y (2009) Large-scale network intrusion detection based on distributed learning algorithm. Int J Inf Secur 8: 25–35.

[5] Beghdad R (2009) Critical study of neural networks in detecting intrusions. Comput Secur. 27: 168–175.

[6] Wu J, Peng D, Li Z, Zhao L, Ling H (2015) Network intrusion detection based on a general regression neural network optimized by an improved artificial immune algorithm. PLoS ONE 10(3): e0120976 doi: 10.1371/journal.pone.0120976 25807466 10.1371/journal.pone.0120976 PMC4373783

[7] Antonatos S, Anagnostakis KG, Markatos EP (2004) Generating realistic workloads for network intrusion detection systems. ACM SIGSOFT Software Engineering Notes 29(1): 207–215.

[8] Cabrera JB, Gosar J, Lee W, Mehra RK (2004) On the statistical distribution of processing times in network intrusion detection. Proceedings of IEEE Conf Decis Control 1:75–80.

[9] Baker ZK, Prasanna VK (2004) Time and area efficient pattern matching on FPGAs. FPGA, 223–232.

[10] Clark CR, Lee W, Schimmel DE, Contis D, Kone M, Thomas A (2005) A hardware platform for network intrusion detection and prevention. Proceedings of Workshop on Network Processors and Applications, 136–145.

[11] Clark CR, Schimmel DE (2003) Efficient reconfigurable logic circuits for matching complex network intrusion detection patterns. Proceedings of International Conference on Field Programmable Logic and Applications, 956–959.

[12] Lee J, Hwang SH, Park N, Lee SW, Sun S, Kim YS (2007) A high performance NIDS using FPGA-based regular expression matching. Proceeding of Symp Appl Comput, 1187–1191.

[13] Aitra A, Najjar W, Bhuyan L (2007) Compiling PCRE to FPGA for accelerating Snort IDS. Proceedings of ACM/IEEE Symposium on Architecture for Networking and Communications Systems, 127–136.

[14] Meiners CR, Patel J, Norige E, Torng E, Liu AX (2010) Fast regular expression matching using small TCAMs for network intrusion detection and prevention systems. Proceeding of USENIX Secur Symp, 8–8.

[15] Sourdis I, Pnevmatikatos D (2004) Pre-decoded CAMs for efficient and high-speed NIDS pattern matching. Proceedings of IEEE Int Symp Field Program Cust Comput Mach, 258–267.

[16] Liu RT, Huang NF, Chen CH, Kao CN (2004) A fast string-matching algorithm for network processor-based intrusion detection system. ACM Transactions on Embedded Computing System 3(3): 614–633.

[17] Bacon DF, Rabbah R, Shukla S (2013) FPGA programming of the masses. Commun ACM 56(4): 56–63.

[18] Scarpazza DP, Villa O, Petrini F (2008) Exact multi-pattern string matching on the Cell/B.E. processor. Comut Front Conf, 33–42.

[19] Schuff DL, Choe YR, Pai VS (2008) Conservative vs. optimistic parallelization of stateful network intrusion detection. Proceedings of International Symposium on Performance Analysis of Systems and Software, 32–43.

[20] Vallentin M, Sommer R, Lee J, Leres C, Paxson V, Tierney B (2007) The NIDS cluster: scalable, stateful network intrusion detection on commodity hardware. Proceedings of International Symposium on Recent Advances in Intrusion Detection, 107–126.

[21] Knuth DE, Morris J, Pratt V (1977) Fast pattern matching in strings. SIAM J Comput 6(2): 127–146.

[22] Boyer RS, Moore JS (1977) A fast string searching algorithm. Commun ACM 20(10): 762–772.

[23] Aho AV, Corasick MJ (1975) Efficient string matching: an aid to bibliographic search. Commun ACM 18(6): 333–340.

[24] Wu S, Manber U. A fast algorithm for multi-pattern searching. Tucson (AZ): University of Arizona, Department of Computer Science; 1994. Report No.: TR-94-17.

[25] Snort.Org [Internet]. Cisco Systems, Inc.; Available: http://www.snort.org.

[26] Jacob N, Brodley C (2006) Offloading IDS computation to the GPU. Proceedings of Computer Security Applications Conference, 371–380.

[27] Huang NF, Hung HW, Lai SH, Chu YM, Tsai WY (2008) A GPU-based multiple-pattern matching algorithm for network intrusion detection systems. Proceedings of International Conference on Advanced Information Networking and Applications, 62–67.

[28] Vasiliadis G, Antonatos S, Polychronakis M, Markatos EP, Iasnnidis S (2008) Gnort: high performance network intrusion detection using graphics processors. Proceedings of International Symposium on Recent Advances in Intrusion Detection, 116–134.

[29] Vasiliadis G, Polychronakis M, Ioannidis S (2011) MIDeA: a multi-parallel intrusion detection architecture. Proceedings of ACM Conference on Computer and Communication Security, 297–308.

[30] Wu C, Yin J, Cai Z, Zhu E, Cheng J (2009) An efficient pre-filtering mechanism for parallel intrusion detection based on many-core GPU Security Technology. Springer Berlin Heidelberg 298–305.

[31] Anagnostakis KG, Antonatos S, Markatos EP, Polychronakis M (2003) E^2^xB: A domain-specific string matching algorithm for intrusion detection. Proceedings of the 18th IFIP International Information Security Conference, 217–228.

[32] Intel Corporation [Internet]. Intel SSE4 programming reference; 2007. Available: http://www.jaist.ac.jp/iscenter-new/mpc/altix/altixdata/opt/intel/vtune/doc/SSE4_Reference.pdf.

[33] Intel Corporation [Internet]. Intel intrinsics guide. Available: https://software.intel.com/sites/landingpage/IntrinsicsGuide/.

[34] Fog A. Instruction tables: lists of instruction latencies, throughputs and micro-operation breakdowns for Intel, AMD and VIA CPUs Denmark (Lyngby): Technical University of Denmark; 2012.

[35] Fatahalian K, Houston M (2008) A closer look at GPUs. Commun ACM 51(10): 50–57.

[36] Nickolls J, Buck I, Garland M, Skadron K (2008) Scalable parallel programming with CUDA. ACM Queue 6(2): 40–53.

[37] Nvidia Corporation [Internet]. NVIDIA CUDA architecture introduction & overview; 2009. Available: http://developer.download.nvidia.com/compute/cuda/docs/CUDA_Architecture_Overview.pdf.

[38] Nvidia Corporation [Internet]. NVIDIA CUDA C Programming Guide; 2015. Available: http://docs.nvidia.com/cuda/pdf/CUDA_C_Programming_Guide.pdf.

[39] OpenMP [Internet]. Available: http://openmp.org.

[40] DEF CON [Internet]. Available: https://www.defcon.org.

[41] Intel Power Gadget [Internet]. Available: https://software.intel.com/en-us/articles/intel-power-gadget-20.

[42] GPU-Z [Internet]. Available: http://www.techpowerup.com/gpuz.

